# Association between Cystic Fibrosis exacerbations, lung function, T2 inflammation and microbiological colonization

**DOI:** 10.1186/s13223-023-00760-z

**Published:** 2023-02-27

**Authors:** Dana Albon, Lijia Zhang, James Patrie, Marieke Jones, Z. Galvin Li, Emily Noonan, Larry Borish

**Affiliations:** 1 Departments of Medicine, University of Virginia School of Medicine, 800546, Charlottesville, VA 22908 USA; 2 Department of Psychiatry, Virginia Commonwealth University, Richmond, VA USA; 3 Public Health Sciences, University of Virginia, Charlottesville, VA USA; 4 Department of Statistics, University of Virginia, Charlottesville, VA USA; 5 Department of Microbiology, University of Virginia, 800546, Charlottesville, VA 22908 USA

**Keywords:** Cystic fibrosis, Type 2 inflammation, Eosinophil, Immunoglobulin E, Pulmonary exacerbation, Fungi, Bacteria

## Abstract

**Background:**

The Cystic Fibrosis Foundation Patient Registry (CFFPR) reports a high prevalence of asthma (34.6%) in people with Cystic Fibrosis (PwCF). While our current understanding of this relationship is limited, a type 2 inflammatory (T2) phenotype has often been identified in CF patients.

**Research question:**

This study aimed to evaluate the relationship between the eosinophilic CF T2 inflammatory phenotype and CF-related pulmonary outcomes and microbiological data.

**Study design and methods:**

We conducted a retrospective chart review of adult patients with CF (18 and older; n = 93) receiving their care at University of Virginia Medical Center adult program from January, 2013 through December, 2018. Data collected included demographic data, CFTR (CF transmembrane conductance regulator) mutation, CF comorbidities, medications, Absolute Eosinophil Counts (AEC) in cells/µL and Immunoglobulin E (IgE) levels in IU/mL.

**Results:**

Of 93 patients screened for study eligibility, 74 were included in the final analysis; 19 patients were excluded due to lack of longitudinal data across the study timeline. Lung function decline correlated with increased AEC (p < 0.001) and IgE (p < 0.001) even when adjusting for covariates: age, gender, presence of *Pseudomonas spp*., MRSA, other bacterial spp., *Aspergillus spp*., and other fungi (p < 0.001). Univariate analysis demonstrated that people with CF who experienced more than 2 exacerbations requiring hospitalizations and/or intravenous antibiotics a year were more likely to have high AEC (p = 0.018). Logistic regression showed that as AEC increases, the probability that the measurement was taken during a CF exacerbation increases (p = 0.0039). A linear mixed model showed that each additional annual exacerbation event increased on average the log IgE by 0.04. (p = 0.015). This finding remained stable in a multivariate model (p = 0.0145). When adjusted for atopy, log IgE increases as the number of exacerbation events increases (p = 0.022). There was no association between AEC and IgE and microbiological colonization.

**Interpretation:**

This study has shown that in CF patients, T2 inflammation based on serum AEC and IgE correlated with pulmonary exacerbations requiring hospitalizations and/or intravenous antibiotics, independent of bacterial airway colonization. In addition, lung function decline correlated with increased IgE and AEC. Further studies are needed to explore these correlations and potential impact on treatment.

**Supplementary Information:**

The online version contains supplementary material available at 10.1186/s13223-023-00760-z.

## Introduction

The Cystic Fibrosis Foundation Patient Registry (CFFPR) reports a high prevalence of asthma (34.6%) in people with Cystic Fibrosis (PwCF) [1]. While our current understanding of this relationship is limited, a type 2 inflammatory (T2) eosinophilic phenotype has often been identified in CF patients [2]. Moreover, medication targeting this phenotype has improved the clinical course of CF patients with T2 inflammation [3].

This type 2 inflammation is characterized by high total IgE antibody titers, specific IgE sensitization and eosinophilia. Also central to this signature are type 2 cytokines (interleukin (IL)-4, IL-5, and IL-13) and type 2 cytokine producing cells (in airway or sputum), such as type 2 innate lymphoid cells [4–6]. CF mouse models have shown an exaggerated IgE response to *Aspergillus fumigatus*, with higher levels of IL-13 and IL-14 [7]. Moreover, naïve CD4 + T cells from CF transmembrane conductance regulator (CFTR) deficient mice produce elevated levels of IL-4 after T cell receptor (TCR) ligation compared to wild-type CD4 + T cells [8]. This T2 signature has also been identified in CF patients prior to and after *Pseudomonas aeruginosa* infection, with a T2 inflammatory phenotype being an independent risk factor for infection [9].

This study aimed to evaluate the relationship between the eosinophilic CF T2 inflammatory phenotype and CF-related pulmonary outcomes and microbiological data. As there are many parameters for T2 inflammation, we focused primarily on absolute eosinophil count (AEC) and IgE levels. We hypothesized that higher levels of AEC and IgE would be associated with poor lung function (FEV1), increased frequency of CF exacerbations, and higher susceptibility to microbiological infections.

## Methods

### Patient population

We conducted a retrospective chart review of adult patients with CF (18 and older; n = 93) receiving their care at University of Virginia Medical Center adult program from January, 2013 through December, 2018. Inclusion criteria were diagnosis of CF, and complete data in our medical system for at least 3 of the 5 years. Overall, 19 patients were excluded from the study due to incomplete data during the study period, due to having less than one annual visit at our center, undergoing lung transplantation during the study period, moving to a different center during the study period, or being new to our center in the period, due to transitioning from pediatric or moving to our center from a different center.

### Data collected

Patient characteristics collected included demographic data (age, sex), CFTR mutation, CF comorbidities (CF-related diabetes, Allergic Bronchopulmonary Aspergillosis (ABPA), Asthma), medications (CFTR modulators, inhaled corticosteroids, inhaled antibiotics, antifungals), Absolute Eosinophil Counts (AEC) in cells/µL (all values available in the electronic medical record during the study period) and total Immunoglobulin E (IgE) levels in IU/mL (average of the patient’s maximal annual IgE counts during the study period) and specific respiratory or fungal Immunoglobulin E panels. Asthma diagnosis was collected in the study data if it was documented by the clinical team in the electronical medical record at any time during the study period. PpFEV1 (percent predicted Forced Expiratory Volume in 1 s) and rate of pulmonary exacerbations (identified as an event that required intravenous therapy and/or hospitalization) were retrospectively collected. The severity of CF disease based on mean ppFEV1 was determined at the end of the study period, in 2018. The CF severity was defined as normal if ppFEV1 was 90% or higher. Mild CF disease was defined as ppFEV1 between 70 and 90%, moderate as ppFEV1 of 40–90% and severe disease if ppFEV1 was below 40% [10]. Mean lung function was defined as mean of the two highest FEV1 level in 2018 at the end of study period. Frequent exacerbations were defined as more than 2 per year. Average AEC was defined as the highest annual AEC level averaged per year of study period. The IgE titers were measured either with annual routine labs as a screening for ABPA, as per CFF chronic care guidelines, or on day one of hospital admissions, prior to initiating antibiotics as per a standardized protocol instituted in 2014 at our center. The rationale of this protocol is to identify early exacerbations related to ABPA/asthma. We propose that elevated AEC during exacerbations is a marker of a T2 phenotype. Highest IgE annual levels were collected for this study when multiple levels were available. The reason for this choice was to eliminate the confounding decrease in AEC and IgE levels seen with steroid use. Given the retrospective limitations of the study, we could not account for oral steroid use during the study period as these are frequently prescribed in our patient population by outside providers, and are not recorded in our electronic medical record. Average AEC was used for univariate analysis, while all AEC values available in the EMR were used for longitudinal statistical analysis and multiple regression analysis. Due to paucity of IgE data, only highest available value per person per year was used for the analysis. The AECs obtained during exacerbations were included in both analyses as we considered them a marker of T2 inflammation regardless of trigger (environmental allergens, antibiotics, or a component of CF-associated inflammation). As standard of care at our institution, with each regular visit and hospitalization, CBC and differential, and bacterial, fungal, and acid-fast bacterial cultures are obtained (if patient could provide good sputum specimens). We specifically collected sputum culture data for *Mycobacteria sppc*, *Methicillin Sensitive and Resistant Staphylococcus Aureus* (MRSA), *Pseudomonas Aeruginosa*, *Stenotrophomonas* spp., *Achromobacter* spp*.*, *Aspergillus Fumigatus* and other *Aspergillus* spp*.*, *Exophiala* spp., *Rasamsonia* spp., and *Scedosporium* spp. Fungal and bacterial presence was defined as detection of a positive fungal or bacterial culture of patient sputum, respectively.

AEC and IgE were considered high if they were above 300 cells/µL and 180 IU/mL respectively. We also grouped together *Exophiala* spp*., Rasamsonia* spp*., and Scedosporium* spp*.* as (E/R/S) due to their small sample size as we conjectured that it would be important to include them in our analyses since those fungal groups are known to cause disease in CF patients. Similarly, *Achromobacter* spp. and *Stenotrophomonas* spp. were grouped together.

This study was approved by our institution’s Institutional Review Board Human Subjects Research (IRB-HSR) committee and followed procedures with ethical standards.

## Statistical analyses

### Univariate analysis

Categorical data are summarized by frequencies and percentages, and continuous scaled data are summarized by the median and interquartile range, and range of the empirical distribution.

#### AEC and IgE quantification description

For the AEC related univariate analyses, the subject-specific AEC data represents the subject’s average of maximum annual AEC values over the study period, while for the IgE related analyses, the subject-specific IgE data represents the subject’s average of maximum annual IgE values over the study period.

#### Average AEC and IgE and lung function

The relationship between AEC and lung function and the relationship between IgE and lung function was examined in two ways. In the first analysis, AEC/IgE served as the outcome variable, and lung function served as the stratification variable, where lung function was stratified into the following ordinal categories: normal, mildly impaired, moderately impaired, and severely impaired based on ppFEV1. The nonparametric Kruskal–Wallis test was used to compare the lung function stratified AEC/IgE empirical distributions. In the second analysis, the ordinal categories for lung function served as outcome variable, and AEC/IgE served as the predictor variable. Ordinal logistic regression (OLR) was used to examine if a higher level of lung function impairment is associated with higher levels of AEC/IgE. Outcome versus predictor association was quantified by the OLR proportional odds ratio test (POR), and proportion odds ratio represented relative difference between odds of having a higher level of lung function impairment between two hypothetical subjects: one with an AEC/IgE value equal to the 75th percentile of the AEC/IgE empirical distribution, and the other with an AEC/IgE value equal to the 25th percentile of the AEC/IgE empirical distribution.

#### Average AEC and IgE and pulmonary exacerbations

The relationship between AEC or IgE and the number of annual pulmonary exacerbations was examined in exactly the same way as the relationships between AEC and IgE lung function. A CF exacerbation was defined as a respiratory exacerbation event that required intravenous antibiotics and/or hospitalization. For both sets of analyses the number of pulmonary exacerbations was ordinally categorized as: 0, 1–2, and > 2 pulmonary exacerbation per year per person. For example, if a person had 3 exacerbations per year in any given year of the study period, he would be included in the analysis as > 2 pulmonary exacerbations.

#### Average AEC and IgE and microbiological infections

AEC and IgE levels were compared between subjects who tested positive and subjects who test negative for: (A) *Mycobacteria spp.,* (B) *Methicillin Resistant Staphylococcus Aureus* (MRSA), (C) *Pseudomonas aeruginosa*, (D) *Stenotrophomonas* spp., (E) *Achromobacter* spp*.*, (F) *Aspergillus fumigatus* and other *Aspergillus* spp*.*, (G) *Exophiala* spp., (H) *Rasamsonia* spp., and (I) *Scedosporium* spp. Per *microbiological* organism, 2 sets of analyses were conducted. One set of analysis compared via the nonparametric two-sample Wilcoxon Rank Sum test the AEC/IgE empirical distributions of those subjects who had positive sputum tests for the *microbiological* organism to the AEC/IgE empirical distributions of those subjects who had negative sputum tests for the *microbiological* organism. The second set of univariate analyses was focused on determining via logistic regression (LR) if the subjects AEC/IgE levels could predict the presence of the *microbiological* organism. The LR odds ratio test compared the odds for testing positive for the *microbiological* organism between a hypothetical subject who has an AEC/IgE level equal to the 75th percentile of the AEC/IgE empirical distribution, relative to a hypothetical subject who has an AEC/IgE level equal to the 25th percentile of the AEC/IgE empirical distribution.

#### Longitudinal data analysis

Longitudinal data were analyzed using mixed effects models with a random effect of patient built in R using the lme4 and lmerTest packages. Two sets of secondary models were built that considered covariates age, gender, ppFEV1, and presence of Pseudomonas spp., MRSA, other bacteria (*Stenotrophomonas spp*., *Achromobacter spp*), Aspergillus spp., or other fungi (*Exophiala spp., Rasamsonia spp., and Scedosporium spp*) and separately, a positive allergen panel (specific respiratory or fungal Immunoglobulin E panels) [11]. AEC and IgE were analyzed after a log-transformation. Linear mixed effects models were built to explain AEC value by whether the measurement was taken during a pulmonary exacerbation, annual variability in AEC by the number of annual exacerbations, annual IgE values by the number of annual exacerbations, and ppFEV1 by AEC and IgE (excluding ppFEV1 as a covariate). One final linear mixed effects model was built to explain AEC values using IgE levels. Poisson mixed models were used to explain the number of annual pulmonary exacerbations using AEC or IgE and using the covariate sets listed above. A linear regression explained variability in IgE by the total number of exacerbations experienced by a patient across the study period and considered the two sets of covariates listed above.

#### Significance level

For all analyses, a two-sided p ≤ 0.05 decision rule was used as the null hypothesis rejection criterion.

## Results

### Baseline demographic and characteristics

Baseline demographics and patient characteristic data are presented in Table 1. Of 93 patients screened for study eligibility, 74 were included in the final analysis; 19 patients were excluded due to lack of longitudinal data across the study timeline. Our sample of patients had a prevalence of CF-related diabetes of 43.2%, ABPA of 8.3%, and physician diagnosed asthma of 82.3%. Our sample of patients had a prevalence of *Pseudomonas aeruginosa* of 70%, MRSA of 32.9%, and *Mycobacteria spp.* of 20.8% over the study period. Notably, when highest AEC per year were averaged over the study period 34 (45.9%) patients had AEC levels above 300 cells/μL. In our cohort, 18.9% of the patients had a maximum IgE level >180 IU/mL. Most of the patients in our sample were on inhaled corticosteroids and CFTR modulators (Table 1).

**Table 1:** Subject characteristics

Characteristic	
Age: mean (SD) [range]	35.1 (10.8) [18, 62]
Female Sex: n (%)	40 (53.3)
delF508 Homozygous: n (%)	44 (59)
CF related diabetes: n (%)	32 (43.2)
ABPA (allergic bronchopulmonary aspergillosis): n (%)	6 (8.3)
Asthma: n (%)	60 (82.3)
*Aspergillus Fumigatus* sensitization (IgE > 0.35 Ku/L): n (%)	17 (23.0)
Any fungal sensitization (IgE > 0.35 Ku/L): n (%)	9 (12.0)
Fungal presence: n (%)	
* Aspergillus spp.*	51 (69.9)
* Exophiala, or Rasamsonia or Scedosporium (ERS)*	23 (31.5)
Bacterial presence: n (%)	
Pseudomonas	51 (69.9)
MRSA	24 (32.9)
* Achromobacter spp. or Stenotrophomonas spp.*	22 (30.1)
Mycobacteria	15 (20.8)
Exacerbations: n (%)	
0 Exacerbations per year	15(20.2)
1–2 Exacerbations per year	42 (57.5)
> 2 Exacerbations per year	17 (23.3)
Medications: n (%)	
CFTR modulators	51 (69.9)
Inhaled corticosteroids	51 (69.9)

### AEC and IgE and lung function

With respect to AEC, there was no detectable shift in the central location of distribution between the 4 different levels of lung function impairment: normal, mild, moderate or severe (p = 0.877). Greater AEC was not a significant predictor of disease severity (POR: 1.24; 95% CI [0.82, 1.88], p = 0.298). With respect to IgE, there was a detectable shift in the central location of distribution between the 4 different levels of lung function impairment (p = 0.049), with the highest median IgE value for the group of patients with moderate lung function impairment. However greater IgE was not a significant predictor of disease severity. (POR: 1.06; 95% CI [0.95, 1.18], p = 0.332).

**Fig. 1:**
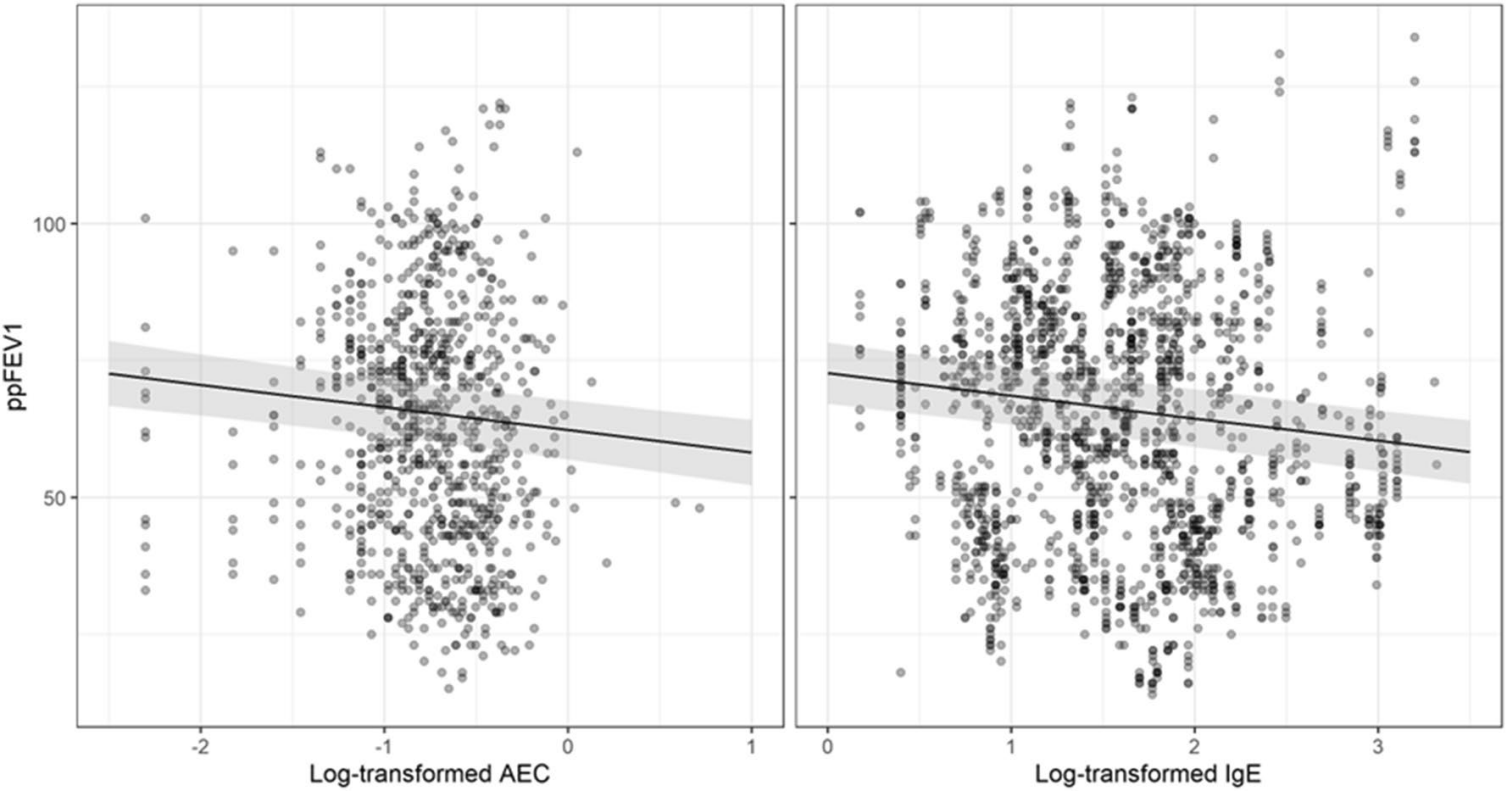
Relationship between lung function (ppFEV1) and AEC and IgE. Image on the left A linear mixed model showed a statistically significant relationship between ppFEV1 decline and log AEC increase (p < 0.001) Image on the right: A linear mixed model showed a statistically significant relationship between ppFEV1 decline and log IgE increase (p < 0.001). ppFEV1 = percent predicted Forced Expiratory Volume in 1 s. AEC = absolute eosinophil count IgE = Immunoglobulin E

A linear mixed model showed a statistically significant negative association between ppFEV1 and log AEC (p < 0.001). This negative association remained statistically significant even when adjusting for age, gender, presence of *Pseudomonas spp.,* MRSA, other bacterial spp., *Aspergillus spp*, and other fungi (p < 0.001). Also in this model, increased age was correlated with a decline in ppFEV1 (p = 0.0396), while there was no statistically significant relationship between ppFEV1 and the other covariates (Fig. 1 left).

A linear mixed model showed demonstrated a statistically significant correlation between ppFEV1 decline and IgE increase (p < 0.001). This correlation remained statistically significant in a multivariate linear mixed model when after adjusting for age, gender, presence of Pseudomonas spp., MRSA, other bacterial spp., Aspergillus spp, and other fungi (p < 0.001). In this model, increasing increased age was also correlated with a decline in ppFEV1 decline (p = 0.04). (Fig. 1 right).

### AEC and IgE and pulmonary exacerbations

**Fig. 2:**
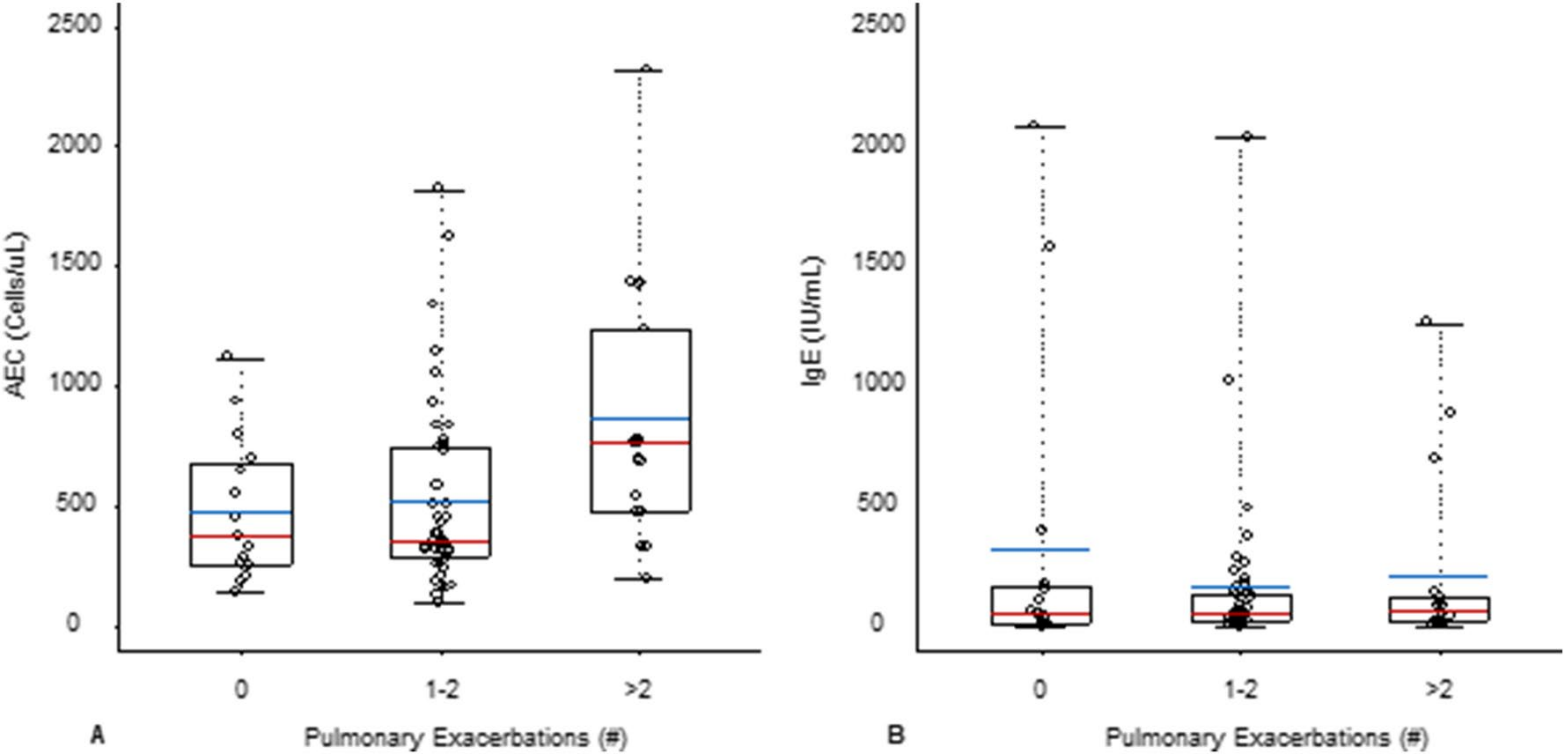
A. Pulmonary exacerbations, AEC and IgE. AEC = Absolute Eosinophil Count; highest annual AEC value averaged per year/per person over the study period was used to calculate median and interquartile range for AEC for each subgroup. *Greater AEC was a significant predictor of greater number of pulmonary exacerbation episodes (POR: 1.88; 95% CI [1.14, 3.08], p = 0.013). B. IgE = Immunoglobulin E; highest annual IgE value averaged per year per person over the study period was used to calculate median and interquartile range IgE for each subgroup

With respect to AEC, there was a detectable shift in the central location of distribution between the 3 different levels of pulmonary exacerbation (p = 0.018) Fig. 2, with the highest median AEC for the group of patients with > 2 pulmonary exacerbations. Greater AEC was a significant predictor of greater number of pulmonary exacerbation episodes (POR: 1.88; 95% CI [1.14, 3.08], p = 0.013). With respect to IgE, there was no detectable shift in the central location of distribution between the 3 different levels of pulmonary exacerbation (p = 0.994). Greater IgE was not a significant predictor of greater number of pulmonary exacerbation episodes (POR: 0.96; 95% CI [0.84, 1.04], p = 0.489).

**Fig. 3:**
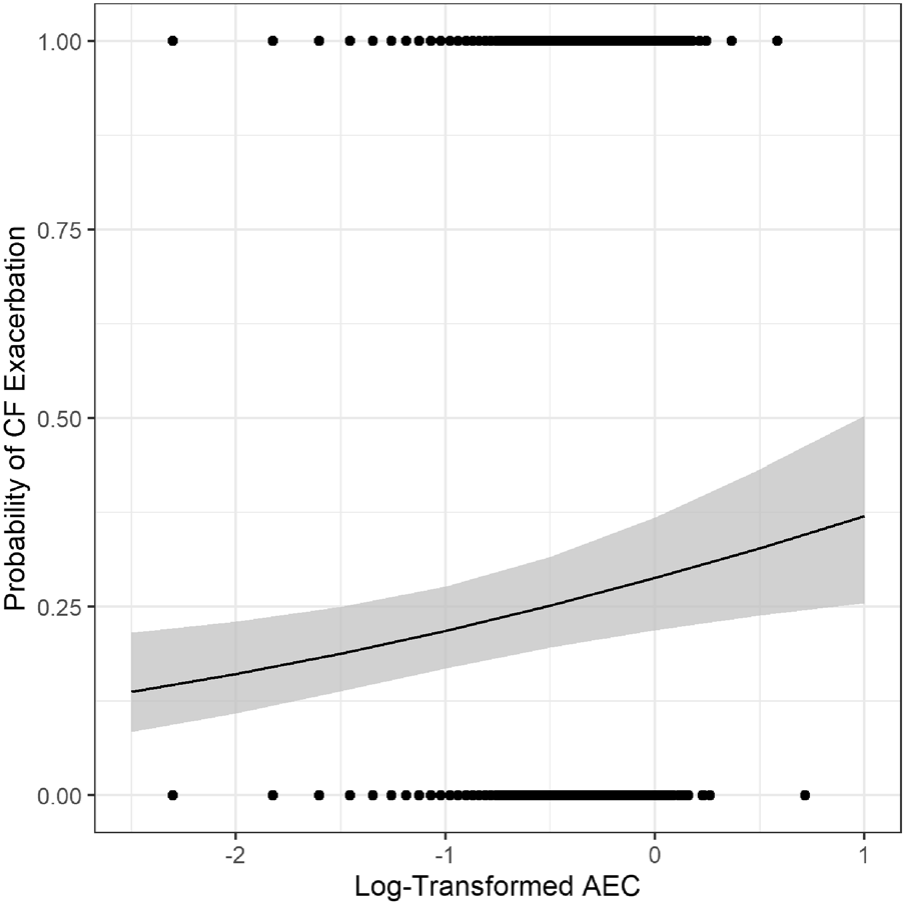
Pulmonary exacerbations and AEC logistic regression: as log AEC increases, the probability that the measurement was taken during a CF exacerbation increases (p = 0.0039) AEC = Absolute Eosinophil Count

A longitudinal model comparing AEC within and outside of exacerbation events showed that log AEC was lower outside exacerbations when compared to levels related to an exacerbation event (p = 0.0035) (Additional file 1: Fig. S1) After adjusting for age, ppFEV1, presence of *Pseudomonas spp*., MRSA, other bacterial spp., *Aspergillus spp*, and other fungi, the effect of CF exacerbation was no longer significant (p = 0.4372). However, presence of a CF exacerbation correlated with ppFEV1 decrease (p = 0.0003). In addition, per each additional annual exacerbation, the annual variance in AEC increased (p < 0.001), an effect that remained significant after adjusting for age, gender, ppFEV1, presence of *Pseudomonas spp*., MRSA, other bacterial spp., *Aspergillus spp.*, and other fungi (p = 0.0001). Logistic regression showed that as log AEC increases, the probability that the measurement was taken during a CF exacerbation increases (p = 0.0039) (Fig. 3). Finally, although Poisson mixed models failed to identify an effect of log AEC on the number of annual exacerbations (p = 0.2248), increasing lung function decreased the number of exacerbations (p = 0.0067) and the presence of *Aspergillus spp.* was associated with more annual CF exacerbations (p = 0.0204).

**Fig. 4:**
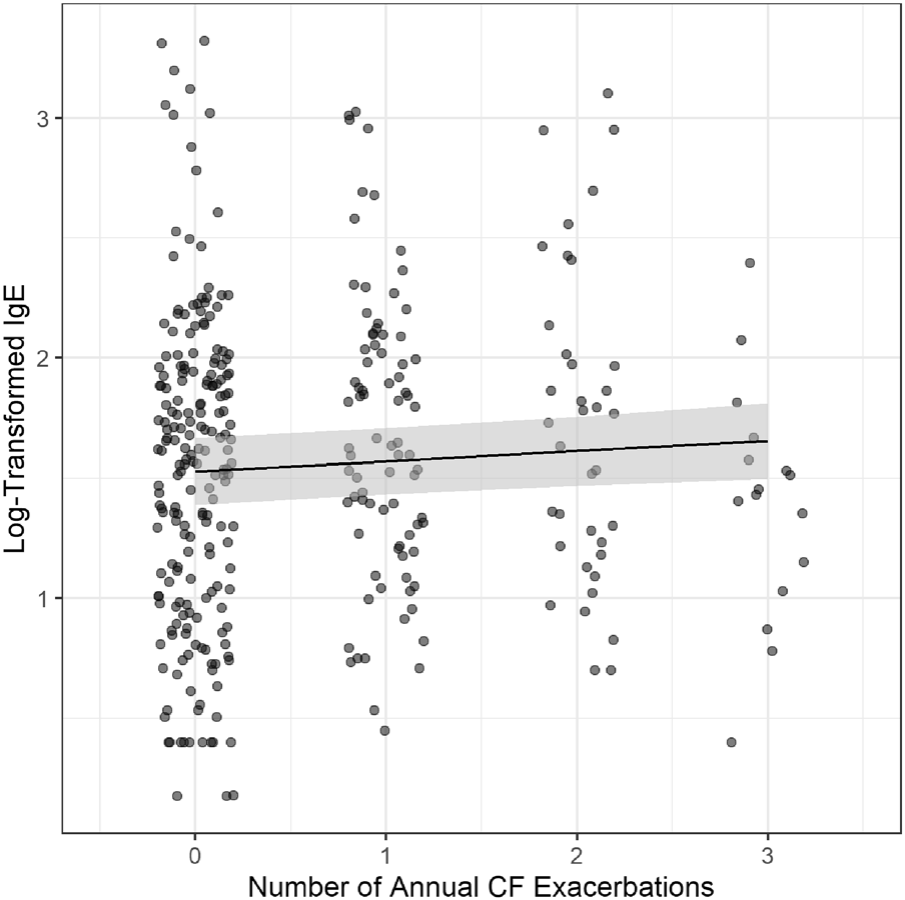
IgE and Pulmonary exacerbations: Increased log IgE correlates with increased numbers of annual CF exacerbations (p = 0.0015). IgE = Immunoglobulin E

A linear mixed model showed that each additional annual exacerbation event increased on average the log IgE by 0.04. (p = 0.015) Fig. 4. This finding remained stable even when adjusting for age, gender, ppFEV1, presence of *Pseudomonas spp*., MRSA, other bacterial spp., *Aspergillus spp.*, and other fungi (p = 0.0145).

**Fig. 5:**
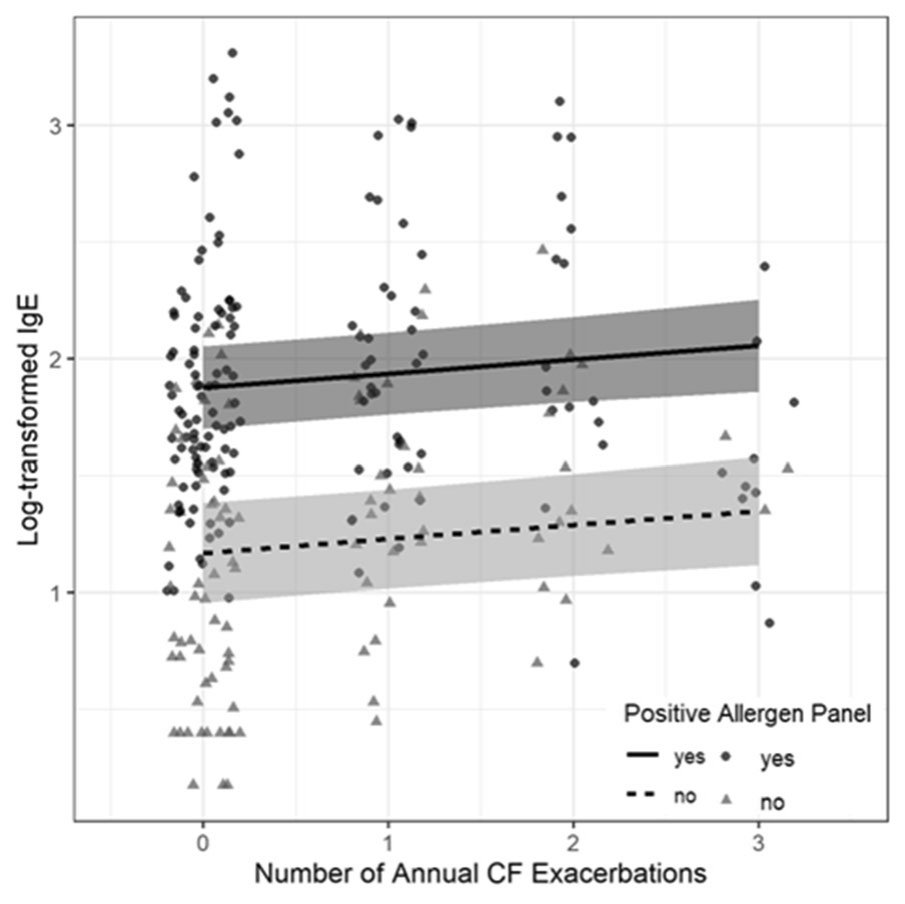
Pulmonary exacerbations and IgE in presence of atopy (positive respiratory allergen or fungus allergen IgE panels) An increase in log IgE is noticed with each additional annual exacerbation (p = 0.0044) and significantly higher log IgE in allergen positive patients (p < 0.001). IgE = Immunoglobulin E

The presence of a positive serum allergen panel further influenced this correlation. A linear mixed model showed an increase in log IgE by 0.707 for each additional annual exacerbation (p = 0.0044) and significantly higher log IgE in allergen positive patients (p < 0.001). While a Poisson mixed model explaining the number of annual CF exacerbations with just log IgE showed no effect of log IgE (p = 0.2263), the model including atopy showed that in the presence of atopy based on positive serum allergen panels, as log IgE increases the exacerbation events increase (p = 0.0222) Fig. 5. The other Poisson mixed model identified presence of *Aspergillus spp.* (p = 0.0003) and decreasing ppFEV1 (p = 0.0110) as significant predictors of the number of CF exacerbations.

### Average AEC and IgE and microbiological fungi infections

Univariate analysis. Empirical distribution numerical summaries are provided in Additional file 1: Table S3. With respect to AEC, there was no shift in the central location of the AEC empirical distribution due to the presence of *Aspergillus,* but there was a marginal upward shift in center location of the AEC empirical distribution due to presence of *Exophiala* or *Rasamsonia* or *Scedosporium* (p = 0.076). AEC was not a predictor of the presence of *Aspergillus* (OR = 0.86; 95% CI [0.52, 1.44], p = 0.570), nor was AEC a predictor of the presence of *Exophiala* or *Rasamsonia* or *Scedosporium* (OR = 1.52; 95% CI [0.91, 2.54], p = 0.105).

With respect to IgE, there was no shift in the central location of the IgE empirical distribution due to the presence of *Aspergillus,* and there was no shift in center location of the IgE empirical distribution due to presence of *Exophiala* or *Rasamsonia* or *Scedosporium*. IgE was not a predictor of the presence of *Aspergillus* (OR = 1.07; 95% CI [0.90, 1.28], p = 0.436), nor was IgE a predictor of the presence of *Exophiala* or *Rasamsonia* or *Scedosporium* (OR = 0.99; 95% CI [0.86, 1.14], p = 0.915).

### Average AEC and IgE and microbiological bacterial infections

Univariate analysis. Empirical distribution numerical summaries are provided in Additional file 1: Table S4A. With respect to AEC, there was no shift in the central location of the AEC empirical distribution due to the presence of *Pseudomonas, MRSA, Achromobacter* or *Stenotrophomanas,* but there was a marginal upward shift in center location of the AEC empirical distribution due to presence of *Mycobacteria* (p = 0.099). AEC was not a predictor of the presence of *Pseudomonas, MRSA, Achromobacter* or *Stenotrophomanas*, or *Mycobacteria* (Table 5B). With respect to IgE, there was no shift in the central location of the AEC empirical distribution due to the presence of *Pseudomonas, MRSA, Achromobacter* or *Stenotrophomanas,* or *Mycobacteria*. IgE was not a predictor of the presence of *Pseudomonas, MRSA, Achromobacter, Stenotrophomanas*, or *Mycobacteria* (Additional file 1: Table S4B)*.*

### Relationship between IgE and longitudinal AEC

A linear mixed effects model was built to explain longitudinal AEC values using annual IgE levels. The model showed a statistically significant inverse correlation between the two variables, as the log IgE increases, the log AEC decreases (p = 0.0269).

## Discussion

Prior to the introduction of highly effective CF modulator therapy, the CF Foundation patient registry report showed that at any given year, approximately 40% of patients had one or more exacerbations a year requiring hospitalization and/or intravenous antibiotics and approximately 10% of this group had 3 or more exacerbations a year. Each year, there is a slight change in the patient groups, some patients do not develop exacerbations annually but every other year or every 3 years and some patients suddenly develop more active disease, and more frequent yearly exacerbation. Following this cohort longitudinally allowed us to include in the analysis more patients with less (1–2 exacerbations, a less active disease) and more frequent exacerbations (more than 2) or more active disease, over a longer period of time. In addition, when a patient develops more than 2 exacerbations a year, our group initiates a frequent exacerbation protocol to identify possible new contributors to the change in phenotype which includes evaluation of ABPA/allergic or atopic phenotype based on serum IgE and AEC levels. Of note, our population has a high exacerbation rate, with 57.5% of our patients having 1–2 exacerbations per year and 24.5% of patients having 3 or more exacerbations per year. While this could be a result of our center’s geographical location it can also be a result of our study period (6 years), which allowed more incidences to be counted than otherwise i.e., even if a patient had only 1 exacerbation in the study period, they would be counted in the 1–2 Exacerbations per year cohort. In addition, our center monitors very closely the AEC and IgE levels with each exacerbation, which most likely led to an increased identification of elevated levels for these two measurements.

As per our hypothesis, higher levels of AEC were associated with more frequent exacerbations, defined as > 2 pulmonary events that required hospitalization and/or IV antibiotics. Our study shows that CF exacerbations increase the AEC levels and corelate with higher AEC variability. The correlation between CF exacerbation and AEC may be explained by exposure to intravenous antibiotics, however the correlation between CF exacerbations and AEC variability is a new finding that is worth further exploration. Based on our study however we cannot draw a conclusion on a cause and effect. It is unclear if exacerbations cause increased T2 inflammation or T2 inflammation predisposes to increased exacerbations.

When accounting for positive allergen panel positivity, as IgE increases, exacerbation events increase. In addition, each annual exacerbation is associated with an increase in IgE and this effect was significantly more pronounced in patients with atopy. This is a new finding and suggests that T2 inflammation may be partially responsible for increased exacerbations. If proven in multicenter, larger study, could lead to a change in CF management with increased use of ICS, and asthma immunological therapies. T2 inflammation leads to reduced expression of antimicrobial proteins, goblet cell hyperplasia and increased mucus production which subsequently can lead to increased bacterial burden, increased respiratory symptoms and subsequentially, increased pulmonary exacerbations [13].

Another novel finding of our study is that longitudinal analysis showed that ppFEV1 decline correlated with increased AEC and IgE even when adjusting for age, gender, presence of *Pseudomonas spp*., MRSA, other bacterial spp., *Aspergillus spp,* and other fungi. Our population is older, with a mean of 35 years, and also has a higher prevalence of *Pseudomonas* (70%), MRSA (32%), and Mycobacteria (21%) than previously reported in other population cohorts. Despite the high infection rates, only 23% of the patients had frequent (> 2) exacerbations/year at any time during the study period. Moreover, neither AEC nor IgE were statistically associated with any bacterial strain. We believe this indicates that the relationship between AEC and pulmonary exacerbations is independent of bacterial presence. While the exact nature of this relationship is unclear, research has shown that eosinophils participate in pulmonary pathology [14]. This could indicate a direct impact of the T2 phenotype on pulmonary structure and performance. Moreover, some of these CF pulmonary exacerbations could have been triggered by eosinophil-mediated changes in mucus production and airway expression of inflammatory mediators.

While bacteria were not correlated with either AEC or IgE, the E/R/S fungi were almost statistically-significantly correlated (p = 0.076) with elevated AEC. We decided to evaluate these fungi despite their low sample size as *Exophiala* spp*, Rasamsonia* spp*, and Scedosporium* spp. have been shown to cause pulmonary disease in CF patients [15, 16]. The lack of statistical significance could be due to grouping the fungi during analyses or can be due to fungi-induced diseases, as well as underpowering due to the small cohort included in our analysis. On univariate analysis, there was no correlation between AEC and IgE and *Aspergillus spp* despite the high prevalence of *Aspergillus* in our population (69%). In the models with covariates, exacerbation rates did not correlate with bacterial colonization but did correlate with *Aspergillus* colonization suggesting that perhaps frequent antibiotic and steroids exposure increases the risk of *Aspergillus* colonization. This is an interesting finding as *Aspergillus* can generate a T2 response and has been associated with ABPA in the CF population [7, 17] observations that warrant further studies.

UVA is situated in Charlottesville, Virginia which is on the asthma belt. Most of our subjects come from rural Virginia areas and work on farms or own farmlands and frequently work with animals and are exposed to multiple allergens. Virginia is a hot-humid, forest-heavy state. Our center’s geographical location and humid weather exposes our patients to a large variety and concentration of pollen and mold allergens. We recognize that a weakness of this study is the utilization of self-reported or physician-diagnosed asthma, and it is possible that the true prevalence of asthma is smaller. To avoid possible bias when using asthma as a variable in the analysis, we chose not to explore this diagnosis. According to the Virginia Department of Health, the 2016 asthma prevalence in Virginia was 8.6%, compared to the national average asthma prevalence of 7.7%. Environmental exposures and/or asthma exacerbations could underlie both correlations between AEC and pulmonary exacerbations and E/R/S respectively. The geographical environment may skew our patient population towards a T2 phenotype. Our patient population is also older with a mean age at study time of 35 which could also be a confounder as there is an increase the risk of sensitization in our patient cohort with increasing age. We found age to be a statistically significant covariate in some models with covariates and, as expected, age correlated with ppFEV1 decline.

There are several limitations to this study and further research is required to better understand the implications of elevated peripheral absolute eosinophils counts in CF disease. We are limited by our study’s retrospective design, unique geographical distribution, and being a single center study with a limited number of patients. Given the retrospective limitations of the study, we could not account for oral steroid use during the study period. These are frequently prescribed in our patient population by outside providers. Highest average AECs were used for univariate analysis to account for this limitation. The AECs obtained during exacerbations were included in the analyses as we think they are a marker of T2 inflammation regardless of trigger (environmental allergens, antibiotics, or idiopathic T2 inflammation itself). Another limitation of our study is the paucity of IgE data. In addition, as mentioned earlier, our study grouped together *Exophiala spp*, *Rasamsonia spp*, and *Scedosporium spp* due to the low number of cases, which may impact findings.

In conclusion, this study has shown that in CF patients, AEC correlated with frequent pulmonary exacerbations requiring hospitalizations and/or intravenous antibiotics, independent of bacterial airway colonization. Treatments that target eosinophilia, such as Mepolizumab (anti-human IL-5 monoclonal antibody), have positive effects in CF patients with T2 inflammation [3]. With this treatment and other biologics that target T2 inflammation in mind, this study’s findings provide an opportunity to address the eosinophilic phenotype in CF patients with current and new immunomodulators approved for asthma treatment as having the potential to lower the health care costs of hospitalization and decrease exposure to intravenous antibiotics.

## Supplementary Information

**Additional file 1: Figure S1**. AEC in exacerbations and during stable outpatient visits. AEC=Absolute Eosinophil Counts. **Figure S2.** Number of exacerbations and IgE levels in the presence and absence of positive allergen panels. **Table S1. **Patient characteristics based on AEC levels. **Table S2. **Patient characteristics based on IgE levels. **Table S3**. T2 Inflammation and Fungal presence. **Table S4**. T2 Inflammation and Bacterial presence.

## Data Availability

Due to risk of HIPAA violation data will not be available outside of UVA.

## Abbreviations

ABPA: Allergic bronchopulmonary aspergillosis
AEC: Absolute eosinophil count
CFFPR: Cystic Fibrosis Foundation Patient Registry
E/R/S: Combination of *Exophiala* spp*.: Rasamsonia* spp*.: and Scedosporium* spp*.*
EMR: Electronic medical record
FEV1: Forced expiratory volume in 1 s: a measurement of lung function
IgE: Immunoglobulin E
MRSA: *Methicillin Resistant Staphylococcus Aureus*
PwCF: Patient with Cystic Fibrosis
T2: Type 2 inflammation
TCR: T cell receptor

## Declarations

### Prior oral and poster presentation

Zhang, L., Borish, L., Patrie, S. & Albon, D. (2021, May 17). Eosinophilic Cystic Fibrosis Is Associated with More Frequent Cystic Fibrosis Exacerbations and Increased Health Care Utilization. [Oral Presentation]. American Thoracic Society, 2021 International Conference, "TP32: Epidemiology, Biomarkers, and Therapy in CF and Non-CF Bronchiectasis"/Charlottesville, VA, USA.
