# Supplementary material for: Association between Cystic Fibrosis exacerbations, lung function, T2 inflammation and microbiological colonization
Source: Allergy Asthma Clin Immunol. 2023 Feb 27;19:15. doi: 10.1186/s13223-023-00760-z (PMC9969710; doi:10.1186/s13223-023-00760-z)
Supplement: Supplementary file 1 — Additional file 1: Figure S1. AEC in exacerbations and during stable outpatient visits. AEC=Absolute Eosinophil Counts. Figure S2. Number of exacerbations and IgE levels in the presence and absence of positive allergen panels. Table S1. Patient characteristics based on AEC levels. Table S2. Patient characteristics based on IgE levels. Table S3. T2 Inflammation and Fungal presence. Table S4. T2 Inflammation and Bacterial presence. [file 13223_2023_760_MOESM1_ESM.docx]

Additional file tables and figures (Appendix)

**Appendix Figure S1**. AEC in exacerbations and during stable outpatient visits. AEC=Absolute Eosinophil Counts.


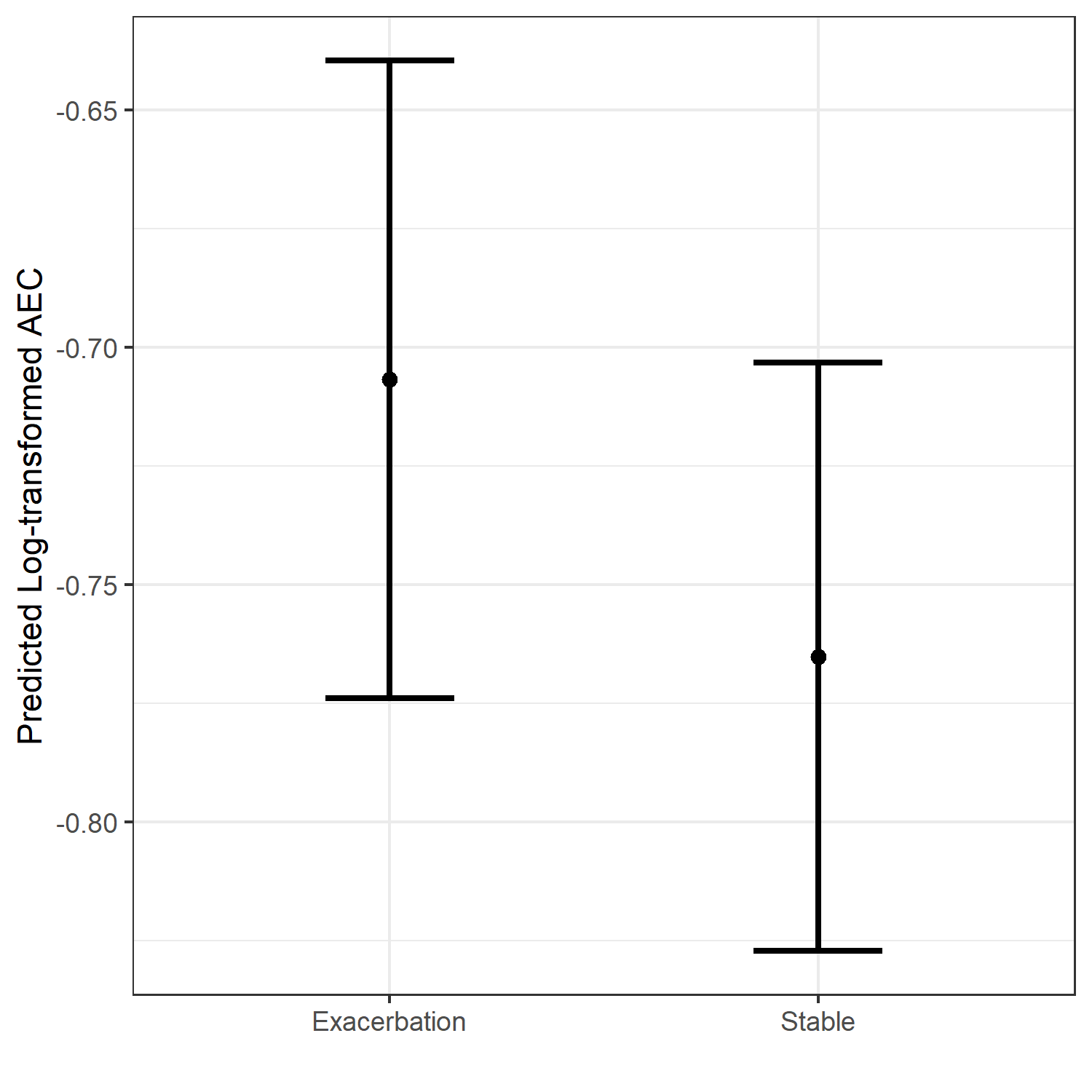


**Appendix Figure S2.** Number of exacerbations and IgE levels in the presence and absence of positive allergen panels

**
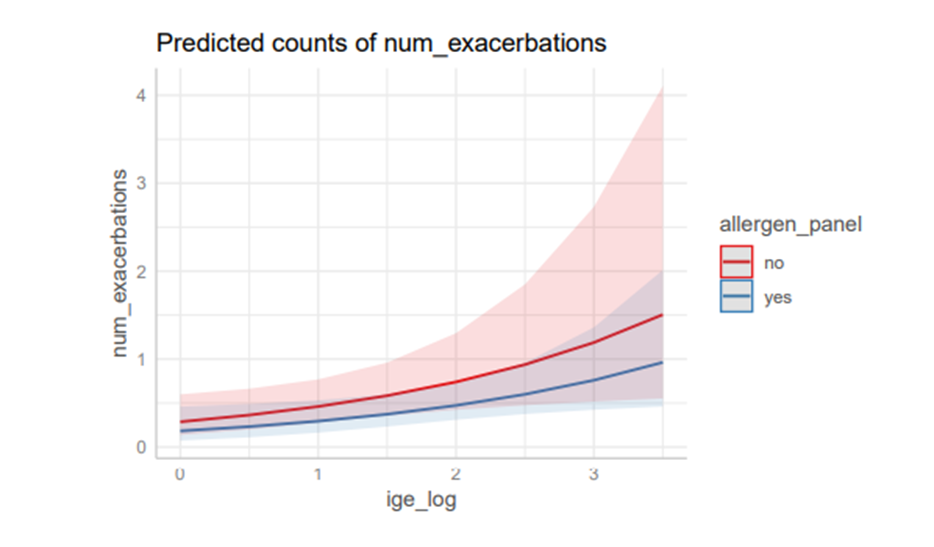
**

**Appendix Table S1.** Patient characteristics based on AEC levels

| Characteristic | AEC < 150 cells/uL,  N = 15(20%) | AEC 150-300 cells/uL  N = 25 (34%) | AEC >= 300 cells/uL,  N = 34(46%) |
| --- | --- | --- | --- |
| **Age on Dec 30, 2017** | 34.9 ± 9.5 | 34.2 ± 9.1 | 33.4 ± 12.4 |
| **Gender** |  |  |  |
| F | 8 (53%) | 12 (48%) | 20 (59%) |
| M | 7 (47%) | 13 (52%) | 14 (41%) |
| **Genotype** |  |  |  |
| Homozygous F508del | 7 (47%) | 15 (60%) | 22 (65%) |
| Heterozygous F508del | 6 (40%) | 8 (32%) | 7 (21%) |
| **ppFEV1** | 73.5 ± 25.8 | 69.9 ± 19.3 | 70.2 ± 25.9 |
| **Frequent Exacerbations** | 3 (20%) | 4 (16%) | 11 (32%) |
| **IgE IU/mL** | 159.2 ± 224.2 | 187.6 ± 465.9 | 260.4 ± 462.1 |
| **Pseudomonas** | 11 (73%) | 19 (76%) | 22 (65%) |
| **MRSA** | 6 (40%) | 11 (44%) | 10 (29%) |
| **Other Bacteria** | 3 (20%) | 9 (36%) | 14 (41%) |
| **Aspergillus spp.** | 6 (40%) | 17 (68%) | 23 (68%) |
| **Other Fungi** | 10 (67%) | 19 (76%) | 21 (62%) |
| **Any Fungi** | 12 (80%) | 25 (100%) | 30 (88%) |
| Mean ± SD; n (%) AEC=absolute eosinophil count, IgE=Immunoglobulin E, MRSA= Methicillin Resistant Staphylococcus Aureus | | | |

**Appendix Table S2. Patient characteristics based on IgE levels**

| Characteristic | IgE < 10,  N = 12(27%) | IgE 10-180,  N = 48(65%) | IgE > 180,  N = 14 (20%) |
| --- | --- | --- | --- |
| **AEC cells/uL** | 322.9 ± 257.5 | 297.3 ± 196.5 | 464.0 ± 337.2 |
| **Age on Dec 30, 2017** | 31.2 ± 7.1 | 34.6 ± 11.0 | 34.4 ± 12.4 |
| **Gender** |  |  |  |
| F | 7 (58%) | 23 (48%) | 10 (71%) |
| M | 5 (42%) | 25 (52%) | 4 (29%) |
| **Genotype** |  |  |  |
| Homozygous F508del | 6 (50%) | 30 (62%) | 8 (57%) |
| Heterozygous F508del | 5 (42%) | 14 (29%) | 2 (14%) |
| **ppFEV1** | 70.2 ± 23.5 | 73.2 ± 23.8 | 63.1 ± 22.6 |
| **Frequent Exacerbations** | 2 (17%) | 13 (27%) | 3 (21%) |
| **Pseudomonas spp.** | 9 (75%) | 32 (67%) | 11 (79%) |
| **MRSA** | 4 (33%) | 16 (33%) | 7 (50%) |
| **Other Bacteria** | 2 (17%) | 16 (33%) | 8 (57%) |
| **Aspergillus spp.** | 8 (67%) | 27 (56%) | 11 (79%) |
| **Other Fungi** | 9 (75%) | 33 (69%) | 8 (57%) |
| **Any Fungi** | 12 (100%) | 42 (88%) | 13 (93%) |
| Mean ± SD; n (%) Mean ± SD; n (%) AEC=absolute eosinophil count, IgE=Immunoglobulin E, MRSA= Methicillin Resistant Staphylococcus Aureus | | | |

**Appendix Table S3**. T2 Inflammation and Fungal presence

| Fungus | Fungus  Present | AEC (Cells/L) | | IgE (UI/mL) | |
| --- | --- | --- | --- | --- | --- |
|  |  | Median [IQR] | p-value† | Median [IQR] | p-value^†^ |
| *Aspergillus* | No | 440.0 [330.0, 877.5] | 0.952 | 83.6 [43.2, 177.8] | 0.220 |
|  | Yes | 478.0 [315.0, 770.0] |  | 52.1 [19.1, 137.5] |  |
| *Exophiala*  *Rasamsonia*  *Scedosporium* | No | 380.0 [265.0, 750.0] | 0.076 | 59.2 [25.1, 138.0] | 0.930 |
|  | Yes | 700.0 [345.0, 805.0] |  | 49.5 [20.6, 161.0] |  |

^†^The p-value is for the nonparametric two-sample Wilcoxon Rank Sum test. AEC=Absolute Eosinophil Count; highest annual AEC value averaged per year/per person over the study period was used to calculate median and interquartile range for AEC for each subgroup. IgE=Immunoglobulin E; highest annual IgE value averaged per year per person over the study period was used to calculate median and interquartile range IgE for each subgroup.

**Appendix Table S4**. T2 Inflammation and Bacterial presence

| **Bacteria** | Bacteria  Present | AEC (Cells/L) | | IgE (UI/mL) | |
| --- | --- | --- | --- | --- | --- |
|  |  | Median [IQR] | p-value^†^ | Median [IQR] | p-value^†^ |
| *Pseudomonas* | No | 550.0 [267.5, 757.5] | 0.818 | 50.4 [20.7, 161.3] | 0.991 |
|  | Yes | 410.0 [320.0, 772.5] |  | 60.4 [23.0, 137.5] |  |
| *MRSA* | No | 450.0 [297.0, 770.0] | 0.876 | 55.2 [23.5, 137.8] | 0.644 |
|  | Yes | 385.0 [317.5, 932.5] |  | 91.4 [19.3, 214.0] |  |
| *Achromobacter* or  *Stenotrphomanas* | No | 410.0 [287.5, 745.0] | 0.255 | 63.9 [24.0, 138.8] | 0.635 |
|  | Yes | 595.0 [327.5, 932.5] |  | 56.0 [19.3, 106.3] |  |
| *Mycobacteria* | No | 390.0 [275.0, 760.0] | 0.099 | 52.4 [20.0, 138.8] | 0.660 |
|  | Yes | 700.0 [360.0, 810.0] |  | 70.1 [45.6, 136.8] |  |
| **(B)**  **Bacteria** | | AEC (Cells/L) | | IgE (UI/mL) | |
|  |  | Odds Ratio^††^  [95% CI] | p-value^†^ | Odds Ratio††  [95% CI] | p-value |
| *Pseudomonas* | | 0.88 [0.53, 1.47] | 0.629 | 1.04 [0.89, 1.21] | 0.642 |
| *MRSA* | | 1.30 [0.79, 2.14] | 0.876 | 1.03 [0.90, 1.17] | 0.667 |
| *Achromobacter* or *Stenotrphomanas* | | 1.23 [0.74, 2.07] | 0.413 | 0.98 [0.85, 1.14] | 0.883 |
| *Mycobacteria* | | 1.27 [0.74, 2.18] | 0.384 | 1.05 [0.92, 1.21] | 0.477 |

^†^p-value of nonparametric two-sample Wilcoxon Rank Sum test

^††^Odds ratios represent odds for bacteria present in someone whose AEC or IgE value is in 75^th^ percentile (770.0Cells/L, 138.8UI/mL), relative to odds for bacteria present in someone who’s in 25^th^ percentile (312.5Cells/L, 22.1UI/mL). Median and interquartile range (IQR) of the AEC and IgE bacteria stratified empirical distributions (A). Odds ratios for examining associations between AEC and IgE and the presence of the bacteria (B).
